# Design, Synthesis, and Evaluation of a Novel Phenanthrene Derivative as a Potential DNA Intercalator

**DOI:** 10.1021/acschembio.6c00169

**Published:** 2026-05-16

**Authors:** Ghada Bouz, Giulia Quaglia, Loredana Latterini, Pavel Barta, Ondřej Jand’ourek, Klára Konečná, Jan Ősterreicher, Lieve Naesens, Leentje Persoons, Jan Storch, Illia Panov

**Affiliations:** † Research Group of Advanced Materials and Organic Synthesis, 86876 Institute of Chemical Process Fundamentals of the Czech Academy of Sciences, Rozvojova 1/135, Prague 6 165 00, Czech Republic; ‡ Faculty of Pharmacy, University Business Academy, Heroja Pinkija 4, Novi Sad 21101, Serbia; § Nano4Light Lab, Department of Chemistry, Biology and Biotechnology, 9309 University of Perugia, via Elce di Sotto 8, Perugia 06123, Italy; ∥ Faculty of Pharmacy in Hradec Králové, Charles University, Hradec Králové 500 03, Czech Republic; ⊥ 54515 KU Leuven, Department of Microbiology, Immunology and Transplantation, Rega Institute, Leuven BE-3000, Belgium

## Abstract

Novel DNA intercalators are crucial for developing new pharmacologically active agents, such as chemotherapeutics, and advanced probes for cell labeling and imaging. Herein, we report the design and two-step synthesis of a novel phenanthrene-based derivative bearing cytosine moieties (PHE-CYT-3,6-TFA), formulated as a trifluoroacetic acid salt to enhance water solubility. *In silico* docking confirmed a favorable binding score of −7.21, predicting intercalation stabilized by π–π stacking and hydrogen bonding. Experimental photophysical characterization confirmed strong DNA interaction, evidenced by a significant decrease in fluorescence quantum yield (from 12% to 5.4%), a spectral blue shift in emission, and changes in fluorescence decay time. The compound was comprehensively screened for *in vitro* antibacterial, antimycobacterial, antifungal, antiviral, and anticancer activities. Crucially, PHE-CYT-3,6-TFA demonstrated excellent biocompatibility, showing nontoxicity toward a noncancerous human cell line (IC_50_ > 50 μM) and no hemolytic activity in an *ex vivo* assay.

## Graphical abstract

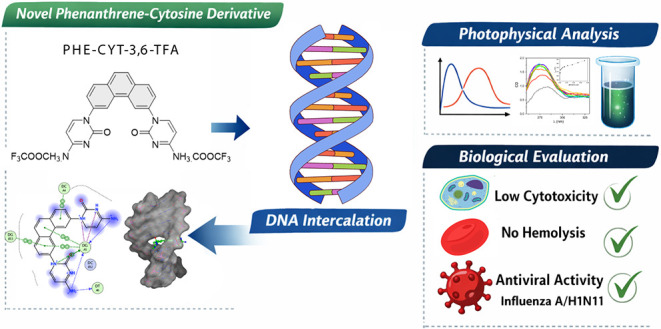

## Introduction and Design Rationale

DNA has a strong affinity for many heterocyclic aromatic compounds, and thus, the design of new entities capable of interacting with DNA has attracted the attention of the scientific community. These interactions can occur through two main mechanisms: either intercalation, where molecules insert themselves between DNA base pairs, or groove binding, where compounds attach to the minor or major grooves of the DNA helix.^1,2^ Experimentally, it is possible to distinguish between intercalators and DNA-groove binders. UV–vis absorption spectroscopy, fluorescence spectroscopy, and circular dichroism (CD) spectroscopy are commonly used for this purpose.^3–8^ In parallel, *in silico* docking studies give insights into the nature of such interactions.

Such interactions are able to significantly alter the structure and function of DNA, influencing gene expression, replication, and repair processes. Pharmacologically, such compounds are used as chemotherapeutic agents, where drugs target DNA to inhibit the proliferation of cancer cells or the replication of pathogens (bacteria, fungi, viruses, etc.).^9,10^ Examples include doxorubicin (an anticancer intercalator), actinomycin D (an antibacterial intercalator), and acriflavine (both an antifungal and an antiviral intercalator). Additionally, understanding these interactions is fundamental in fields such as molecular biology and genetics, as it provides insights into cellular processes that, in turn, play a role in discovering new therapeutic approaches. Applications of such compounds go beyond this and extend to cell labeling and imaging. These compounds, often organic fluorescent dyes or probes, alter the fluorescence and photophysical properties of DNA upon interaction, enabling high-resolution imaging techniques such as fluorescence microscopy and confocal microscopy.^11,12^ Upon interacting with DNA, such compounds may cause changes in the absorption bands, fluorescence quantum yield, and fluorescence lifetime.^13^ These DNA-binding compounds are indispensable in both basic research and clinical diagnostics, providing critical insights into cellular function, genetic regulation, and the molecular fingerprints of various diseases.

In this study, we designed a DNA intercalator bearing two cytosine moieties (PHE-CYT-3,6-TFA). Cytosine is one of the building blocks in the genetic materials of living organisms (DNA and RNA). Beyond its natural biological role, cytosine is widely explored in medicinal chemistry aimed at developing chemotherapeutics against cancer and viral infections.^14–17^ In cancer treatment, such compounds compete with natural nucleosides, thereby interfering with the DNA synthesis of cancer cells and preventing their growth and proliferation. Clinically available examples include cytarabine, which is used primarily for treating leukemia (Figure 1). In the field of antiviral therapy, cytosine analogues suppress the replication of viruses by incorporating into viral DNA or RNA. Zalcitabine and emtricitabine are used for HIV, cidofovir for cytomegalovirus, and lamivudine is used for both hepatitis B and HIV treatment (Figure 1).

**Figure 1:**
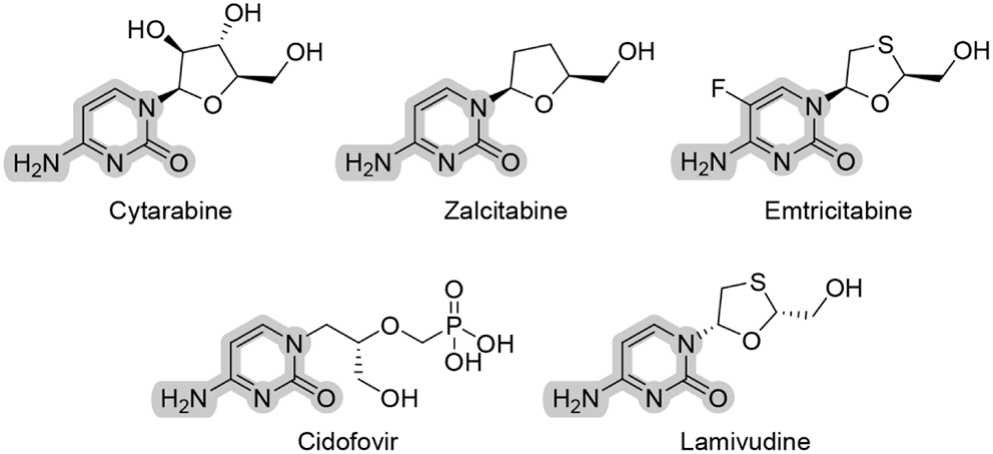
Chemical structures of selected cytosine-containing pharmacologically active compounds. Cytosine is shown in gray.

In this work, we chose the phenanthrene ring as the backbone to bridge the two cytosine moieties (Figure 2a). The rigid nature of phenanthrene contributed to the planarity of our title compound, PHE-CYT-3,6-TFA. A well-known example of a phenanthrene-based DNA intercalator is ethidium bromide, which is used as a cellular dye (Figure 2b). To rationalize our design, we first performed an *in silico* docking experiment. The interaction with DNA via an intercalation mechanism was investigated experimentally. The biological activity of the title compound was explored, evaluating its antiproliferative, antibacterial, antimycobacterial, antifungal, and antiviral properties. Finally, we assessed the potential toxicity in normal, noncancerous human cells and the ability of PHE-CYT-3,6-TFA to induce hemolysis of human red blood cells (RBCs).

**Figure 2:**
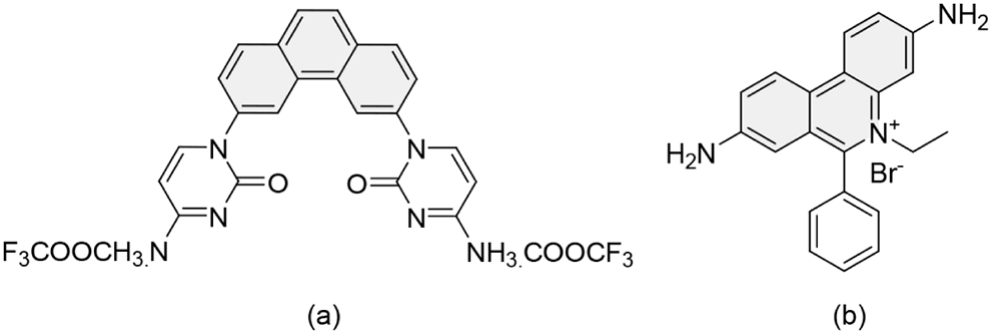
The chemical structures of (a) the title compound PHE-CYT-3,6-TFA and (b) ethidium bromide. The phenanthrene ring is highlighted in gray.

## Materials and Methods

### *In Silico* Modeling

*In silico* calculations were performed in Molecular Operating Environment (MOE) 2022.09 (Chemical Computing Group Inc., Montreal, QC, Canada) under the Amber10:EHT force field. The ligand was prepared using MOE’s built-in function to predict the dominant protonation state at pH 7. The model of DNA (PDB ID: 1N37) was downloaded from the RCSB Protein Data Bank (PDB). The model was prepared using MOE’s QuickPrep functionality with default settings, which included the calculation of partial charges, adding hydrogens, and restrained minimization to an RMS gradient of 0.01 kcal·mol^–1^·Å^–1^. The docking was performed with default MOE settings: docking stage-Placement: Triangle Matcher, score: London dG, retain 50 poses; Refinement stage-Rigid receptor, score: GBVI/WSA dG, retain 5 poses. The ligand site was defined as the coordinate of the crystallographic ligand – Anthracycline Respinomycin D Intercalation Complex.

### General Information

Commercially available reagent-grade materials were used as obtained from Merck (Darmstadt, Germany) and Fluorochem (Derbyshire, United Kingdom). All solvents were of reagent grade and used without any further purification, except tetrahydrofuran, which was distilled from sodium benzophenone ketyl. Melting points were determined using the Santiago KB T300 melting point apparatus (Czech Republic) and are uncorrected. TLC was carried out using silica gel 60 F254-coated aluminum sheets, and compounds were visualized with UV light (254 and 366 nm). Column chromatography was performed using Biotage HPFC systems Isolera One (Biotage, Uppsala, Sweden) with prepacked flash silica gel columns. ^1^H and ^13^C­{^1^H} NMR spectra were recorded using a Bruker Avance spectrometer at 400 MHz (^1^H NMR) and 101 MHz (^13^C NMR). Chemical shifts (δ) are reported in parts per million (ppm) and referenced to residuals of CDCl_3_ (δ = 7.26 and 77.00 ppm, respectively) or DMSO-*d*_6_ (δ = 2.50 and 39.52 ppm, respectively). The coupling constants (*J*) are given in hertz (Hz) with the corresponding multiplicity (s = singlet, d = doublet, t = triplet, m = multiplet). For exact mass measurement, the spectra were internally calibrated using Na-formate or APCI-TOF tuning mix. APCI high-resolution mass spectra were measured in positive mode using a micrOTOF QIII mass spectrometer (Bruker) and were determined by the software Compass Data Analysis. Elemental analyses were performed on a Flash 2000 Organic Elemental Analyzer (Thermo Fisher).

### Synthesis

#### Preparation of Diiodophenanthrene (Procedure A)

6.72 g of 3,6-dibromophenanthrene (20 mmol, 1 equiv) was dissolved in 1000 mL of dry tetrahydrofuran (THF) under argon and cooled down to −78 °C in a dry ice bath. *tert*-Butyllithium (*tert-*BuLi) 25.9 mL (1.7 M, 44.0 mmol, 2.2 equiv), was added slowly dropwise, and the mixture was stirred for an hour. Afterward, 11.2 g of iodine (44 mmol) was added at once, and the reaction was stirred for an additional 1 h. Then the ice bath was removed, and the mixture was left to stir for an additional hour at ambient temperature. The volatiles were then evaporated under reduced pressure, after which 500 mL of dichloromethane (DCM) was added. The solution was washed with a saturated solution of sodium sulfite (Na_2_SO_3_), followed by water and brine, and dried with anhydrous magnesium sulfate (MgSO_4_). The organic layer was evaporated under reduced pressure, and the resulting solid was crystallized from heptane to yield 279.5 g (65%) of the desired product. Refer to Scheme 1.

**Scheme 1:**
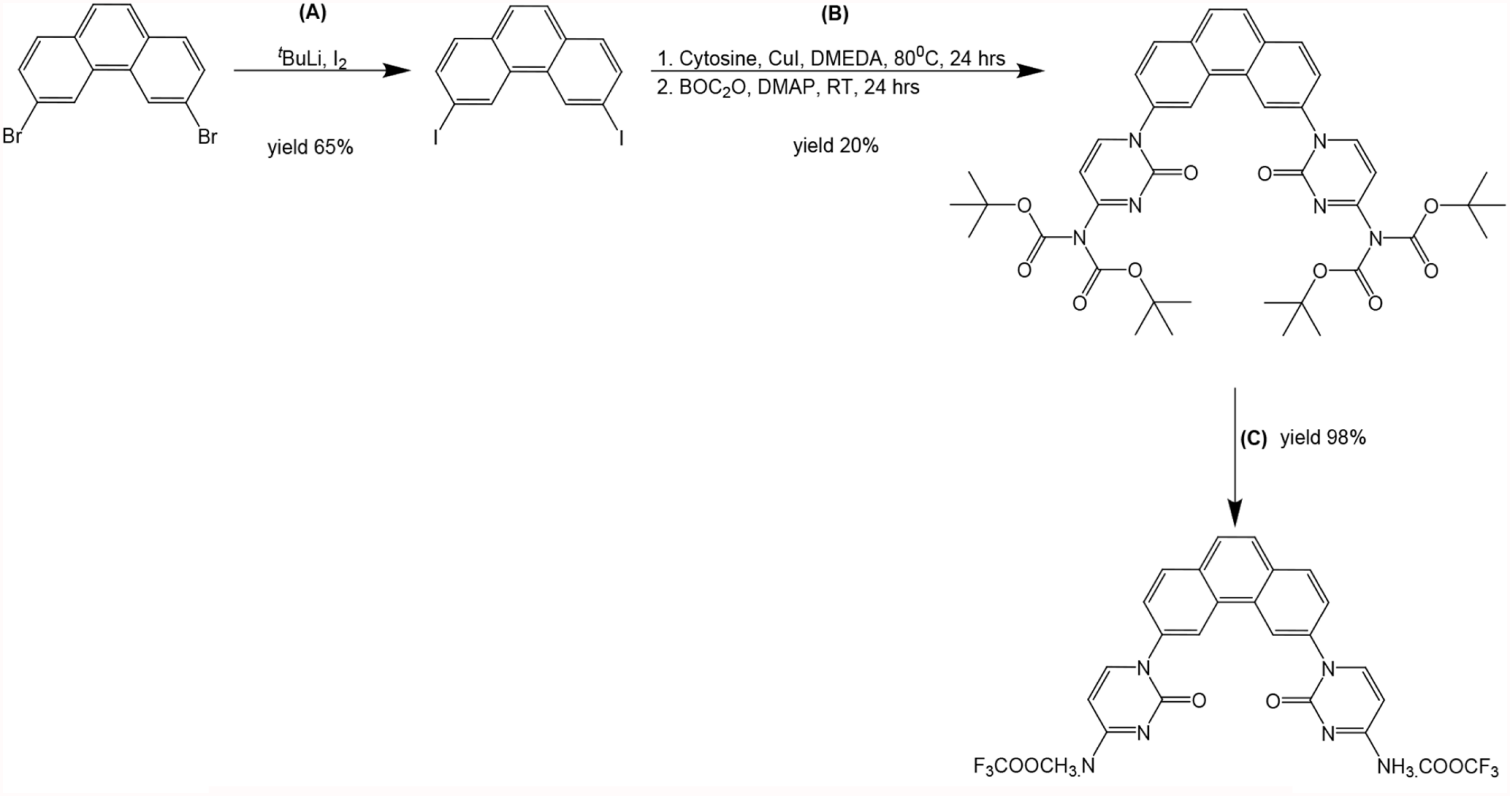
Stepwise Synthesis of Title Compound PHE-CYT-3,6-TFA

#### Preparation of PHE-CYT-3,6-Boc (Procedure B)

In a 25 mL Schlenk flask, 860 mg of 3,6-diiodophenanthrene (2 mmol) was mixed with cytosine (6 mmol, 666 mg) in the presence of CuI (0.1 mmol, 19 mg), DMEDA (0.2 mmol, 22 μL), and K_2_CO_3_ (6 mmol, 828 mg) in 10 mL of dry *N*-methylpyrrolidone. The reaction mixture was stirred under an argon atmosphere and heated to 80 °C for 24 h. The reaction solution changed color from blue to beige over time. Then, the reaction mixture was brought to RT, and 9.0 g di-*tert*-butyl dicarbonate (41 mmol) and 100 mg of DMAP were added to the mixture. The reaction continued for another 24 h at 25 °C. Upon completion, 100 mL of brine was added, and the reaction mixture was extracted with EtOAc (3 × 100 mL). The combined organic layers were dried over anhydrous magnesium sulfate (MgSO_4_), filtered, and the solvents were evaporated under reduced pressure. Finally, the crude product was purified using flash chromatography with methyl *tert*-butyl ether/petroleum ether (MTBE/PE) as the solvent (70/30), yielding 318.8 mg of the desired product (20%). The reaction steps were repeated for scale-up. Refer to Scheme 1.

#### Preparation of Final Compound PHE-CYT-3,6-TFA (Procedure C)

600 mg (0.75 mmol) of the Boc-protected compound was dissolved in 10 mL of DCM, and 10 mL of trifluoroacetic acid was added to the solution at ambient temperature. The reaction was stirred at RT for 3 h. Volatiles were removed under reduced pressure. The final obtained solid was washed with diethyl ether, yielding 98% (267.8 mg). See Scheme 1.

### Analytical Data

#### Di-iodophenanthrene

##### 3,6-Diiodophenanthrene

Yellow solid: mp 224–225 °C; ^1^H NMR (CDCl_3_, 400 MHz): δ 8.90 (s, 2H), 7.87 (d, 2H, *J* = 8.3 Hz), 7.67 (s, 2H), 7.59 (d, 2H, *J* = 8.4 Hz). ^13^C­{^1^H} NMR (CDCl_3_, 101 MHz): δ 135.8, 131.8, 131.0, 130.6, 130.0, 127.0, 92.9.IR: 3092, 3063, 2968, 1903, 1570, 1492, 1131, 870 cm^–1^; HR ESI MS: calculated [M + H]^+^ for C_14_H_8_I_2_ 430.8788, found 430.8791. Anal. Calcd for C_14_H_8_I_2_: C, 39.10; H, 1.88. Found: C, 39.27; H, 2.01.

#### Final Compound bis-Boc

##### Di-*tert*-butyl­(phenanthrene-3,6-diylbis­(2-oxo-1,2-dihydropyrimidine-1,4-diyl))­bis­((*tert*-butoxycarbonyl)­carbamate)

White powder: mp 132–140 °C; ^1^H NMR (CDCl_3_, 400 MHz): δ 8.57 (d, 2H, *J* = 2.1 Hz), 8.00 (d, 2H, *J* = 8.5 Hz), 7.83 (s, 2H), 7.81 (d, 2H, *J* = 7.4 Hz), 7.66 (dd, 2H, *J* = 8.5, 2.0 Hz,), 7.22 (s, 2H, *J* = 8.5 Hz), 1.60 (s, 36H). ^13^C­{^1^H} NMR (CDCl_3_, 101 MHz): δ 162.9, 154.5, 149.5, 148.1, 138.7, 131.8, 130.3, 129.9, 127.4, 125.2, 120.4, 96.7, 85.1, 27.71. IR: 2986, 3891, 1965, 1674, 1495, 1382, 1278, 1261, 1147, 874 vs cm^–1^; HR ESI MS: calculated [M + H]^+^ for C_42_H_49_N_6_O_10_ 797.3505, found 797.3501. Anal. Calcd for C_42_H_48_N_6_O_10_: C, 63.30; H, 6.07; N, 10.55. Found: C, 63.07; H, 6.15; N, 10.61.

#### Final Compound TFA Salt (PHE-CYT-3,6-TFA)

##### 1,1’-(Phenanthrene-3,6-diyl)­bis­(4-aminopyrimidin-2­(1*H*)-one) trifluoroacetate

White powder: mp >300 °C; ^1^H NMR (DMSO-*d*_6_, 400 MHz): δ 9.10–8.95 (bs, 2H), 8.88 (d, 2H, *J* = 2.1 Hz), 8.40–8.29 ()­bs, 2H), 8.19 (d, 2H, *J* = 8.5 Hz), 8.12 (d, 2H, *J* = 7.5 Hz), 8.03 (s, 2H), 7.77 (dd, 2H, *J* = 8.5, 2.0 Hz), 6.11 (d, 2H, *J* = 7.5 Hz). ^13^C­{^1^H} NMR (CDCl_3_, 126 MHz, dimer/monomer mixture): δ 172.9, 172.4, 168.2, 168.1, 161.0, 159.1 (q, 1C, *J* = 33.6 Hz), 150.1, 150.0, 148.8, 137.7, 132.0, 130.2, 130.2, 128.0, 126.5, 116.9 (q, 1C, *J* = 295.8 Hz), 94.51. IR: 3300–2600 vbs, 1678, 1557, 1434, 1221, 1183, 864, 740 cm^–1^; HR ESI MS: calculated [M + H]^+^ for free base C_22_H_17_N_6_O_2_ 397.1408, found 397.1403. Anal. Calcd for C_26_H_18_F_6_N_6_O_6_: C, 50.01; H, 2.91; N, 13.46. Found: C, 50.30; H, 3.09; N, 13.70.

### Photophysical Characterization

Absorption spectra were recorded by the UV–vis Spectrophotometer Shimadzu UV-1900i in the wavelength range of 200–800 nm with a resolution of 1 nm on PHE-CYT-3,6-TFA in DMSO (5 μM) and DNA from calf thymus water solution (8 μg/mL). Photoluminescence spectra were collected by the spectrofluorometer Edinburgh FS5, equipped with a 150W xenon lamp. The luminescence decay times τ_F_, were measured using a spectrofluorometer based on the single-photon counting method, excited with a pulsed LED at 294 nm and collecting the signal at 380 nm. Circular dichroism spectra were recorded using a Jasco J-810 Spectropolarimeter (Jasco Corporation, Tokyo, Japan) in the spectral range of 190–600 nm. The elaboration of the spectra was performed using CDNN software. All spectrometer measurements were conducted using a 1 cm quartz cell.

### *In Vitro* Cytotoxicity against HepG2, A498, HK-2, PC-3, SKOV3, and U87 MG Cell Lines

The human hepatocellular liver carcinoma cell line HepG2 was purchased from the Health Protection Agency Culture Collections (ECACC, Salisbury, UK). The remaining cell cultures used are commercially available from the American Type Culture Collection (ATCC, Manassas, VA, USA). Cell lines were tested for mycoplasma contamination upon their delivery. The 50% inhibitory concentration (IC_50_) values were determined from the dose–response curves in GraphPad Prism 10 (GraphPad Software, Boston, MA, USA). Viability (%) was plotted as a function of concentration (log values) and fitted to a sigmoidal curve, in which the half-maximal inhibitory concentration value was determined, representing the concentration of a compound required for 50% inhibition. A full description of the method can be found in the Supporting Information.

### *In Vitro* Antibacterial Activity Screening

The microdilution broth method was used according to EUCAST, with minor modifications (European Committee for Antimicrobial Susceptibility Testing [EUCAST] of the European Society of Clinical Microbiology and Infectious Diseases).^18^ Results were expressed as MIC, compared with standards of gentamicin (GEN) and ciprofloxacin (CIP). A full description of the method can be found in the Supporting Information.

### *In Vitro* Antimycobacterial Activity Screening

Testing was performed using the Microplate Alamar Blue Assay (MABA)^19^ and according to the EUCAST protocol,^20^ where results were expressed as MIC in μg/mL in comparison with isoniazid (INH), rifampicin (RIF), and ciprofloxacin (CIP) as standards. A full description of the method can be found in the Supporting Information.

### *In Vitro* Antifungal Activity Screening

A microdilution broth method was performed according to EUCAST protocols, with minor modifications.^21,22^ Results were expressed as MIC, compared with the standards amphotericin B (AMB) and voriconazole (VRC). A full description of the method can be found in the Supporting Information.

### *In Vitro* Antiviral Activity Screening

The compound was broadly tested for antiviral activity using cytopathic effect (CPE) reduction assays in (i) human embryonic lung (HEL299) fibroblast cells infected with herpes simplex virus type 1 or human coronavirus-229E or -OC43; (ii) Madin-Darby canine kidney (MDCK) cells infected with influenza virus (A/H1N1, A/H3N2, or B); (iii) African green monkey kidney VeroE6 cells infected with yellow fever virus, Zika virus, Sindbis virus, or Semliki Forest Virus; and (iv) human HEp-2 cells infected with respiratory syncytial virus. Suitable reference compounds were included, i.e., remdesivir, chloroquine, ribavirin, zanamivir, rimantadine, brivudin, and dextran sulfate. CPE was quantified, depending on the virus, on days 3 to 6 post-infection using the colorimetric MTS cell viability assay. This method also enabled the assessment of the compound’s cytotoxic effect in mock-infected cell cultures.

### Employment of *Ex Vivo* Human Red Blood Cell Hemolysis Assay

Blood samples from human volunteers were centrifuged (1000 × *g*) for 10 min, the supernatants were discarded, and the pellets were washed three times with Hartmann’s solution. The final pellet was diluted 1:7 (v/v) with Hartmann’s solution. Then, 0.5 mL of cell suspensions were incubated with the title compound at final concentrations corresponding to the range from 3.906 to 1000 mM. After incubation at 37 °C for 1 h, the cell suspension was centrifuged, and the supernatant was carefully collected. The amount of hemoglobin released from red blood cells (RBCs) into the supernatant was monitored by measuring the absorbance at 405 nm (4, 5) using a spectrophotometer (Synergy HTX Multimode Reader, BioTek, USA). A full description of the method can be found in the Supporting Information.

## Results and Discussion

### *In Silico* Docking Experiment

In order to rationalize the design of PHE-CYT-3,6-TFA, we performed *in silico* docking to evaluate the interactions with DNA base pairs. Our docking poses were consistent with the prediction of an intercalative mechanism, characterized by the insertion of the planar phenanthrene core between adjacent base pairs. This behavior is in agreement with well-established structure–activity relationships, where extended planar aromatic systems are a prerequisite for efficient π–π stacking with nucleobases.^2^ The ligand achieved a score of −7.21 kcal·mol^–1^, indicating a moderately strong binding affinity, consistent with values typically reported for classical DNA intercalators such as doxorubicin and ethidium bromide, which generally fall within the range of −6 to −10 kcal·mol^–1^ depending on the docking protocol and DNA model.^23,24^ Ligand stabilization occurred through two ionic bonds between the positively charged nitrogen and guanidine, three hydrogen bonds formed by positively charged amine functional groups, one π-cation bond, and four π–π interactions (Figure 3). This combination suggests a hybrid binding mode, where classical intercalation is reinforced by electrostatic and hydrogen-bonding contributions. Similar dual binding behavior has been described for cytosine-containing ligands and nucleobase analogues, where hydrogen bonding enhances sequence selectivity and binding stability.^2,25,26^

**Figure 3:**
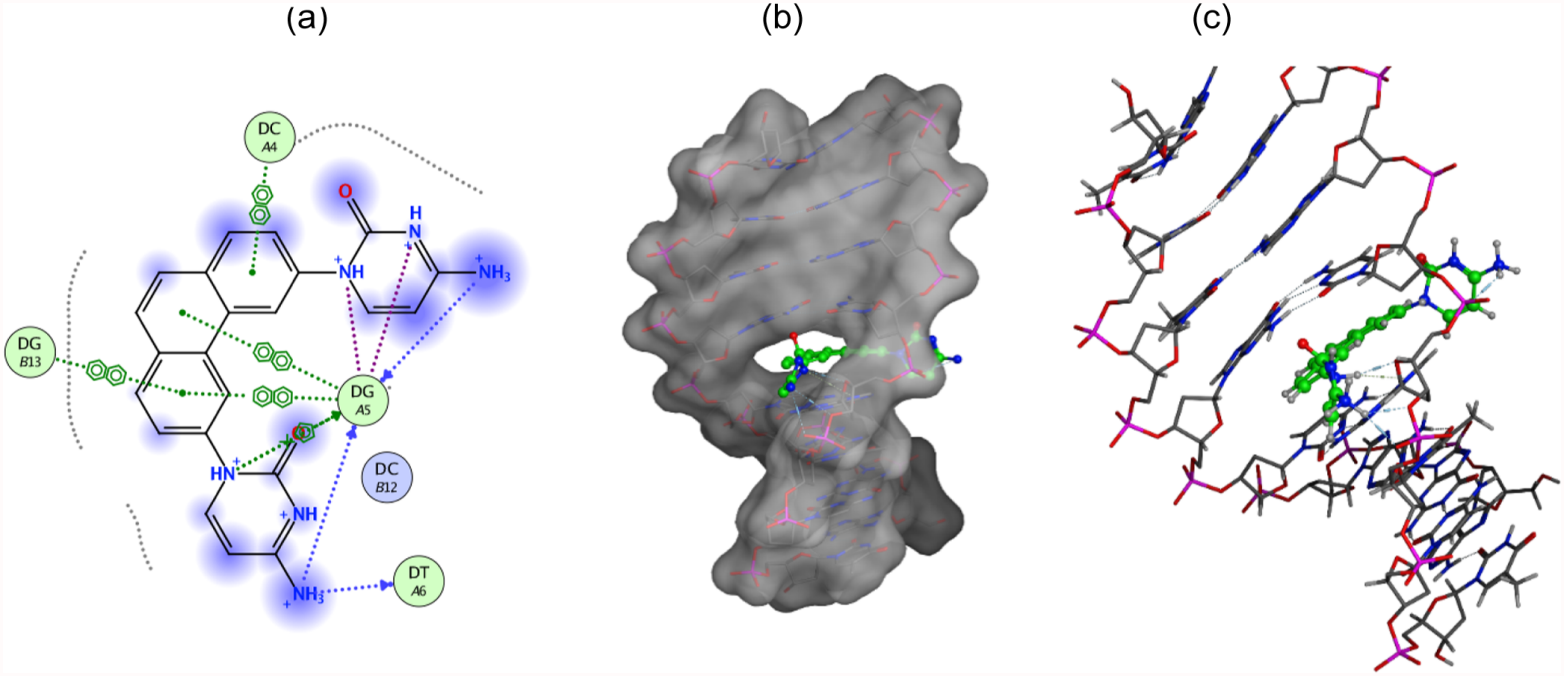
Interaction diagram of the title compound with DNA base pairs. (a) 2D binding pose showing intercalation with DNA bases, highlighting stabilization via positively charged amines and π–cation interactions. (b) Predicted 3D model illustrating the intercalative insertion of the compound between DNA base pairs. (c) Close-up of the intercalating site, highlighting the planar alignment of the phenanthrene core within the base stacks. The docking score was −7.21, supporting the intercalative binding mode.

### Chemistry

PHE-CYT-3,6-TFA was obtained after a two-step synthesis designed to introduce cytosine moieties onto the phenanthrene scaffold while preserving the planarity required for intercalative binding. In the first step, lithium–halogen exchange of 3,6-dibromophenanthrene, followed by iodination, afforded the reactive 3,6-diiodophenanthrene in good yield (65%), providing a suitable intermediate for coupling with an aromatic nucleophile. Subsequent copper-catalyzed C–N cross-coupling with cytosine enabled the addition of two heterocyclic units at positions 3 and 6 of the phenanthrene core, generating the Boc-protected intermediate (yield 20%). This step confirmed the feasibility of simultaneously introducing two nucleobase fragments onto the rigid aromatic system. Final deprotection with trifluoroacetic acid cleanly produced the target compound PHE-CYT-3,6-TFA as its trifluoroacetate salt in excellent yield (98%) as white crystals, improving aqueous solubility and enabling subsequent photophysical and biological evaluation.

### Evidence of Interaction with DNA

The absorption of PHE-CYT-3,6-TFA in DMSO exhibits three major features at 260, 285, and 310 nm, as reported in Figure S1 in Supporting Information. In the same Figure S1, the spectrum of DNA in a water solution is reported (black line), from which the UV absorption at 260 nm is displayed. Changes in the absorption spectra arise upon the addition of DNA to PHE-CYT-3,6-TFA solutions, supporting the hypothesis of interactions.

As expected, the intensity of the band at 260 and 285 nm increases, but it is noteworthy that the intensity of the band at 310 nm decreases and the optical density at longer wavelengths becomes more pronounced, suggesting the presence of an interaction between DNA and the organic molecule (Figure 4a). The increase in absorption likely occurs because the intercalating molecules interact with the π-electron system of the DNA base pairs, increasing the molecule’s ability to absorb light. Additionally, the stacking interactions between the intercalating molecule and DNA base pairs can enhance the molecule’s π–π* transitions, contributing to increased absorption. Moreover, the blue shift of the band from 287 to 283 nm (Figure S2) is an indication that the interaction with DNA might occur via intercalation, with the reduction of freedom of movement of the π electrons, and the consequent reduction of π–π* transition probability.^27^ The presence of an isosbestic point at 293 nm suggests the conversion of the free compound into DNA-associated species. However, the impact of solvent changes upon the addition of aqueous DNA aliquots has to be considered; indeed, upon adding water volumes to PHE-CYT-3,6-TFA (in Figure 4a black dotted line), an increment in the absorption above 350 nm is detectable, probably due to the increasing polarity of the environment.^28^ Fluorescence excitation spectra are generally more accurate tools for monitoring the evolution of the absorption spectra of emitting species. Differences are observed in the excitation spectra: upon the addition of neat water, a decrease in intensity is appreciated, but the shape of the band is unaffected. On the other hand, in the presence of DNA, a broadening at longer wavelengths is observed (Figure 4b). Simultaneously to these changes, significant alterations in the fluorescence spectrum appear upon the addition of DNA (Figure 4c). The emission band shifts to shorter wavelengths, and its intensity decreases. This blue shift in the fluorescence spectrum upon DNA intercalation suggests that the fluorophore is experiencing a more rigid, less polar, and hydrophobic environment due to its insertion between DNA base pairs. This is a commonly observed characteristic in many DNA-binding dyes, such as acridine orange and proflavine.^29,30^ At DNA concentration higher than 0.851 μg/mL a shoulder at 340 nm appears, whose intensity increases with DNA concentration. This observation supports the occurrence of interactions between PHE-CYT-3,6-TFA and DNA, able to modify the electronic configuration of the dye.^31^ The hypothesis is also confirmed by the UV–vis absorption enhancement in the range between 350 nm and 450 nm in the presence of higher concentrations of DNA. In fact, shoulders or new peaks in the UV–vis spectrum, particularly in the 300–400 nm range, are often associated with organic intercalators that have extended π-conjugated systems.

**Figure 4:**
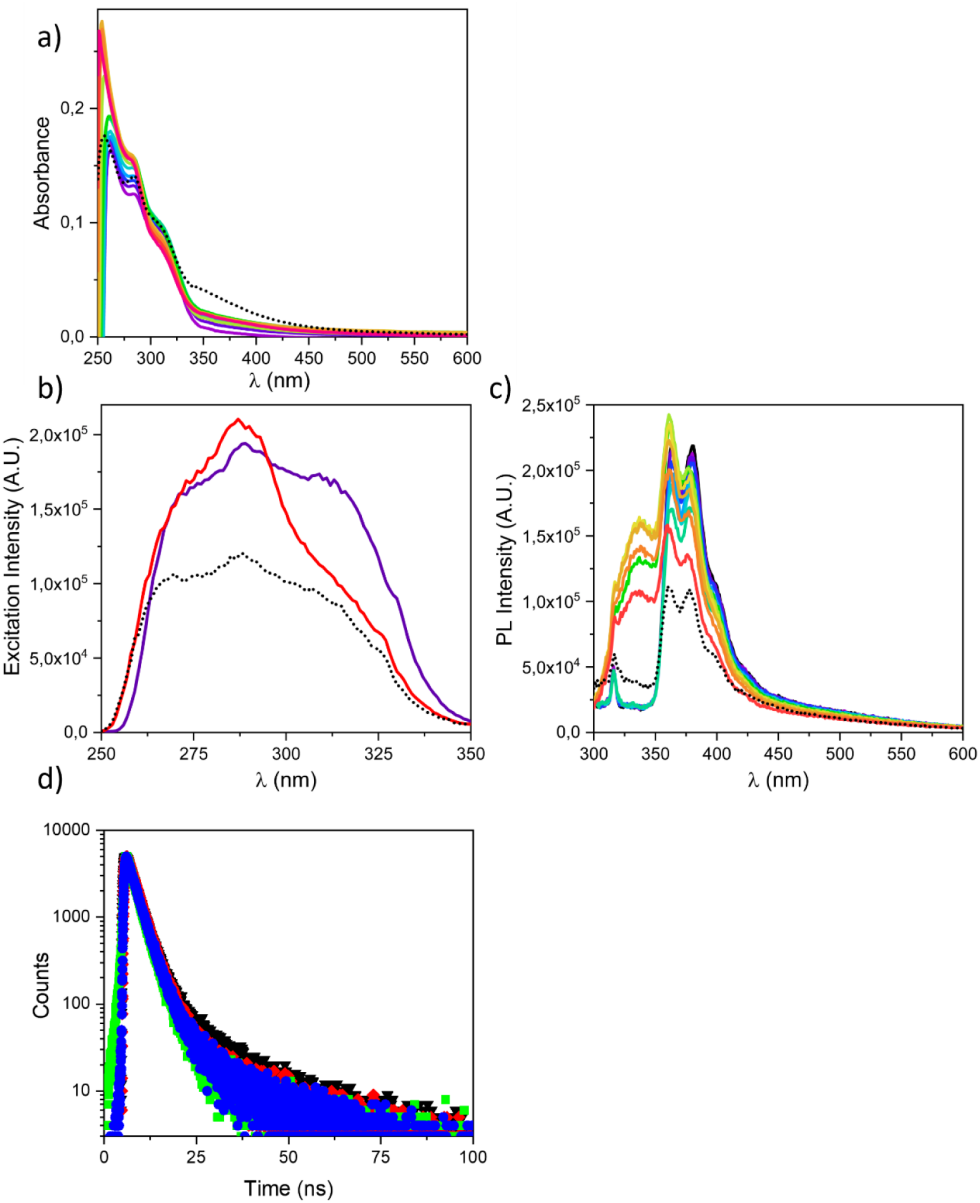
a) Absorption spectra of PHE-CYT-3,6-TFA in DMSO measured with different concentrations of DNA aqueous solution (range: 0.019–3.211 μg/mL). b) Excitation (λ_em_ = 365 nm) of PHE-CYT-3,6-TFA in DMSO (violet), with DNA aq. solution (red), and with water (black). c) Emission (λ_ex_ = 270 nm) spectra measured with different concentrations of DNA aq. solution. Black dotted line: absorption (a) and emission (c) of PHE-CYT-3,6-TFA with water. d) Decay kinetics of PHE-CYT-3,6-TFA in DMSO before (in black) and after the addition of DNA (0.851 μg/mL in green and 3.211 μg/mL in blue) and water (red).

The fluorescence quantum yields determined in the absence and presence of DNA, as well as with water, are listed in Table 1. Upon interaction with DNA, the quantum yield of PHE-CYT-3,6-TFA is halved, and the same trend is observed after the addition of water without DNA (3.4%). However, in the presence of DNA, a band at a shorter wavelength appears. This decrease in quantum yield during intercalation suggests that a significant fraction of the absorbed energy is being lost through nonradiative processes and that the PHE-CYT-3,6-TFA excited state is being stabilized by the DNA environment due to the rigidity of PHE-CYT-3,6-TFA upon binding, which restricts its rotational freedom.

**Table 1:** Luminescence Quantum Yield of PHE-CYT-3,6-TFA in the Presence of Different Concentrations of DNA Strand

Sample	Φ_PL_
PHE-CYT-3,6-TFA in DMSO	12%
PHE-CYT-3,6-TFA in DMSO + DNA (3.211 μg/mL)	5.4%
PHE-CYT-3,6-TFA in DMSO + H_2_O	3.4%

The time-correlated single photon-counting (TCSPC) measurements of PHE-CYT-3,6-TFA in DMSO have been performed in the absence and presence of DNA (Figure 4d). In general, all decays could be very well reproduced when performing a global biexponential analysis. The shorter time constant is found to be around 3 ns, while the longest one is around 14 ns. Upon the addition of DNA at a concentration of 0.851 μg/mL, the fluorescence decay slightly shortens; as the concentration of DNA increases, the kinetics trend is confirmed (Table 2) and the effect is more evident on the long living component. The detected decrease in decay time can be justified by quenching processes between the intercalator and DNA bases. On the other hand, upon the addition of different amounts of neat water to PHE-CYT-3,6-TFA in DMSO, the decay time is not modified, suggesting that the changes observed in the presence of DNA are due to the intercalation process.

**Table 2:** Decay Times of PHE-CYT-3,6-TFA in DMSO-Free Solution and after the Addition of Water and DNA Exciting at 294 nm and Collecting the Emission at 380 nm

Sample	τ (ns)
PHE-CYT-3,6-TFA in DMSO	τ_1_ = 3.0 ns (82%)
	τ_2_ = 14.5 ns (18%)
PHE-CYT-3,6-TFA in DMSO + H_2_O	τ_1_ = 3.0 ns (86%)
	τ_2_ = 13.7 ns (14%)
PHE-CYT-3,6-TFA in DMSO + DNA 0.851 μg/mL	τ_1_ = 3.0 ns (75%)
	τ_2_ = 13.1 ns (25%)
PHE-CYT-3,6-TFA in DMSO + DNA 3.211 μg/mL	τ_1_ = 2.9 ns (85%)
	τ_2_ = 12.9 ns (15%)

For the circular dichroism (CD) measurements, a native DNA aqueous solution was prepared. The spectrum, reported in Figure S3 in Supporting Information, displays a negative signal at 245 nm and a positive one at 275 nm, representative of right-handed ds-DNA.^32^ The band at 245 nm is attributed to DNA helicity, and the band at 275 nm to base stacking.^33^ In order to evaluate the effect of the interaction between the phenanthrene derivative and DNA, the CD spectra of a DNA aqueous solution were collected in the presence of different concentrations of the organic compound and DMSO. The results, illustrated in Figure 5, point out that the increased hydrophobicity of the environment due to the presence of DMSO is responsible for the significant decrease in the intensity (around 21%) of the peak at 275 nm. Moreover, the intermolecular interaction between DNA and PHE-CYT-3,6-TFA causes a red shift of the band at 275 nm (see the plot in the inset of Figure 5). The data suggest that during the interaction with PHE-CYT-3,6-TFA, the DNA maintained its specific right-handed helical conformation, as the CD bands at 275 nm almost retained their original shape.^34^

**Figure 5:**
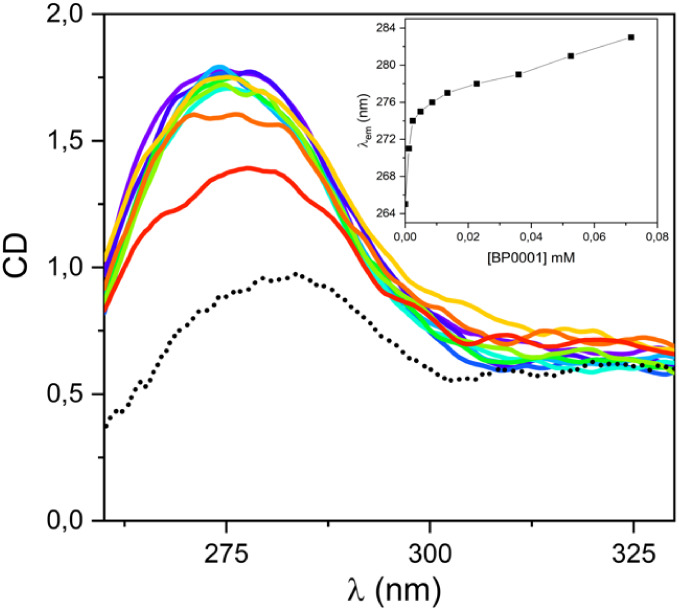
Circular dichroism of DNA aqueous solution (0.29 mg·mL^–1^) in the absence (dark violet line) and in the presence of different amounts of PHE-CYT-3,6-TFA (range: 0.0012–0.0717 mM, blue to red lines) and DMSO (black dotted line). Inset graph: maximum emission wavelength vs PHE-CYT-3,6-TFA molar concentration plot.

### *In Vitro* Cytotoxicity

As stated earlier in the introduction, DNA intercalators may exert antiproliferative activity that makes them promising anticancer agents. Therefore, we explored the *in vitro* cytotoxic activity of our title compound in human epithelial kidney carcinoma cells A-498, human prostate adenocarcinoma cells PC-3, human ovary adenocarcinoma cells SK-OV-3, human glioblastoma cells U-87 MG, and hepatocellular carcinoma cell line HepG2. *In vitro* activity was expressed as IC_50_. As shown in Table 3 below, PHE-CYT-3,6-TFA had no *in vitro* antiproliferative activity up to the highest tested concentration (at higher concentrations, the compound started to precipitate in the testing media). The cytotoxicity of the title compound was also explored in normal, noncancerous human embryonic lung cells HEL 299. Again, the title compound was shown to be safe, with an IC_50_ value over 50 μM.3

**Table 3:** In vitro Cytotoxicity Profile of the Title Compound (PHE-CYT-3,6-TFA) and Its Boc-Protected Intermediate (bis-Boc) across Various Human Cancer and Noncancerous Cell Lines^a^ ^b^

Cmpd.	A-498	PC-3	SK-OV-3	U-87 MG	Hep2	HEL 299
PHE-CYT-3,6-TFA	≥100	≥25	≥100	≥50	>50	>50
Phe-Cyt-3,6-Boc	≥250	≥250	≥250	≥250	na	na

a IC_50_ values are expressed in μM.

b na = not applicable.

### *In Vitro* Anti-Infective Activity Screening

The final compound was evaluated for its anti-infective activity against a panel of pathogens of clinical importance. These included bacteria, namely *Staphylococcus aureus*, methicillin-resistant *Staphylococcus aureus* (MRSA), *Staphylococcus epidermidis*, *Enterococcus faecalis*, *Escherichia coli*, *Klebsiella pneumoniae*, *Acinetobacter baumannii*, and *Pseudomonas aeruginosa*; mycobacteria, namely *Mycolicibacterium smegmatis*, *Mycolicibacterium Aurum*, *Mycobacterium avium*, *Mycobacterium kansasii*, and *Mycobacterium tuberculosis* H37Ra; and fungi, namely *Candida albicans*, *Candida krusei*, *Candida parapsilosis*, *Candida tropicalis*, *Aspergillus fumigatus*, *Aspergillus flavus*, *Lichtheimia corymbifera*, and *Trichophyton interdigitale*. The final compound did not exert significant activity against any of the tested microbes.

The final compound was also screened for its antiviral activity against different DNA and RNA viruses, such as HCoV-229E, HCoV-OC43, Influenza H1N1, Influenza H3N2, Influenza B, RSV A Long, HSV-1 KOS, Yellow Fever Virus 17D, Zika Virus MR766, Sindbis Virus AR-339, and Semliki Forest Virus Original, where it was shown to be inactive. The only exception was the Influenza A/H1N1 virus, for which we measured a 50% effective concentration (EC_50_) of 26 μM. Although this value is quite high compared to standards [ribavirin (EC_50_ = 11 μM); zanamivir (EC_50_ = 0.8 μM); rimantadine (EC_50_ = 7 μM)], the result is intriguing since the influenza virus is unique among RNA viruses in performing its RNA synthesis in the nucleus. This process relies on a “panhandle” double-stranded RNA structure that is formed by the 3′- and 5′-termini of the viral genome and might perhaps be disrupted by an intercalating agent.

### Employment of *Ex Vivo* Human Red Blood Cell Hemolysis Assay

To evaluate the safety profile of the compound and its initial compatibility with systemic administration, an *ex vivo* human red blood cell (RBC) hemolysis assay was conducted. This assay serves as a crucial preliminary test for assessing cytotoxic effects on human erythrocytes, which are highly sensitive indicators of membrane integrity disruption.^35,36^ Compared to the negative control (unexposed cells), the statistical analysis revealed that concentrations below 1000 μM have no significant impact on the cytoplasmic membrane of human red blood cells, and the title compound is considered to be safe and compatible for systemic administration. Refer to Figure S4 in Supporting Information.

## Conclusion

In conclusion, we successfully designed, synthesized, and characterized a novel phenanthrene-based compound bearing two cytosine moieties as a potential DNA intercalator. *In silico* docking confirmed its intercalative nature, positioning the molecule between DNA base pairs with a favorable binding score of −7.21, stabilized by predicted π–π stacking and hydrogen bonding. Comprehensive photophysical studies, including UV–vis, fluorescence spectroscopy, and circular dichroism, strongly suggest that the dye–DNA interaction mechanism is driven by a mixed binding mechanism, i.e., intercalation between the bases and electrostatic interactions between the cationic group and the negatively charged phosphate groups in the DNA. Furthermore, the compound exhibited excellent biocompatibility, demonstrating low cytotoxicity in noncancerous cells (IC_50_ > 50 μM) and nonhemolytic properties in *ex vivo* red blood cell assays, which supports its safety profile for potential biomedical applications. Collectively, these findings highlight PHE-CYT-3,6-TFA as a structurally interesting and biologically safe DNA-interacting agent. Its confirmed ability to interact with DNA makes it a promising candidate for further study in the broader context of DNA-targeting small molecules, particularly as a fluorescent probe or a scaffold for drug development.

## Supplementary Material



## Data Availability

The original contributions presented in this study are included in the article/Supporting Information. Further inquiries can be directed to the corresponding author.

## Notes

Supplementary Figures S1–S4. Detailed experimental procedures (PDF)

## References

[1] Prabhakar P., Kayastha A. M. (1994). Mechanism of DNA-Drug Interactions. Appl. Biochem. Biotechnol..

[2] Strekowski L., Wilson B. (2007). Noncovalent Interactions
with DNA: An Overview. Mutat. Res., Fundam.
Mol. Mech. Mutagen..

[3] Sirajuddin M., Ali S., Badshah A. (2013). Drug–DNA Interactions
and Their Study by UV–Visible,
Fluorescence Spectroscopies and Cyclic Voltametry. J. Photochem. Photobiol., B.

[4] Berova N., Bari L. D., Pescitelli G. (2007). Application
of Electronic Circular
Dichroism in Configurational and Conformational Analysis of Organic
Compounds. Chem. Soc. Rev..

[5] Nordén B., Kurucsev T. (1994). Analysing DNA Complexes by Circular and Linear Dichroism. J. Mol. Recognit..

[6] Chang Y.-M., Chen C. K.-M., Hou M.-H. (2012). Conformational
Changes in DNA upon
Ligand Binding Monitored by Circular Dichroism. Int. J. Mol. Sci..

[7] Passeri R., Aloisi G. G., Elisei F., Latterini L., Caronna T., Fontana F., Sora I. N. (2009). Photophysical
Properties
of N-Alkylated Azahelicene Derivatives as DNA Intercalators: Counterion
Effects. Photochem. Photobiol. Sci..

[8] Viola G., Latterini L., Vedaldi D., Aloisi G. G., Dall’acqua F., Gabellini N., Elisei F., Barbafina A. (2003). Photosensitization
of DNA Strand Breaks by Three Phenothiazine Derivatives. Chem. Res. Toxicol..

[9] Palchaudhuri R., Hergenrother P. J. (2007). DNA as
a Target for Anticancer Compounds: Methods to
Determine the Mode of Binding and the Mechanism of Action. Curr. Opin. Biotechnol..

[10] Mišković K., Bujak M., Baus Lončar M., Glavaš-Obrovac L. (2013). Antineoplastic
DNA-Binding Compounds: Intercalating and Minor Groove Binding Drugs. Arh. Hig. Rada. Toksikol..

[11] Anil A., Chaskar J., Pawar A. B., Tiwari A., Chaskar A. C. (2024). Recent
Advances in DNA-Based Probes for Photoacoustic Imaging. J. Biotechnol..

[12] Wetmur J. G. (1991). DNA Probes:
Applications of the Principles of Nucleic Acid Hybridization. Crit. Rev. Biochem. Mol. Biol..

[13] Kel O., Fürstenberg A., Mehanna N., Nicolas C., Laleu B., Hammarson M., Albinsson B., Lacour J., Vauthey E. (2013). Chiral Selectivity
in the Binding of [4]­Helicene Derivatives to Double-Stranded DNA. Chem. - Eur. J..

[14] Choi W. J., Chung H.-J., Chandra G., Alexander V., Zhao L. X., Lee H. W., Nayak A., Majik M. S., Kim H. O., Kim J.-H., Lee Y. B., Ahn C. H., Lee S. K., Jeong L. S. (2012). Fluorocyclopentenyl-Cytosine with
Broad Spectrum and Potent Antitumor Activity. J. Med. Chem..

[15] Chua G. N. L., Wassarman K. L., Sun H., Alp J. A., Jarczyk E. I., Kuzio N. J., Bennett M. J., Malachowsky B. G., Kruse M., Kennedy A. J. (2019). Cytosine-Based TET
Enzyme Inhibitors. ACS Med. Chem. Lett..

[16] Krečmerová M., Otmar M. (2012). 5-Azacytosine Compounds In Medicinal Chemistry: Current Stage And
Future Perspectives. Future Med. Chem..

[17] Scaglione F., Berrino L. (2012). Cytosine Deoxyribonucleoside
Anti-HIV Analogues: A
Small Chemical Substitution Allows Relevant Activities. Int. J. Antimicrob. Agents.

[18] European Committee for Antimicrobial Susceptibility Testing (EUCAST) of the European Society of Clinical Microbiology and Infectious Diseases (ESCMID) Determination of minimum inhibitory concentrations (MICs) of antibacterial agents by broth dilution. Clin. Microbiol. Infect. 2003, 9(8), ix–xv. 10.1046/j.1469-0691.2003.00790.x. 11168187

[19] Franzblau S. G., Witzig R. S., McLaughlin J. C., Torres P., Madico G., Hernandez A., Degnan M. T., Cook M. B., Quenzer V. K., Ferguson R. M., Gilman R. H. (1998). Rapid, Low-Technology MIC Determination
with Clinical *Mycobacterium Tuberculosis* Isolates
by Using the Microplate Alamar Blue Assay. J.
Clin. Microbiol..

[20] Schön T., Werngren J., Machado D., Borroni E., Wijkander M., Lina G., Mouton J., Matuschek E., Kahlmeter G., Giske C., Santin M., Cirillo D. M., Viveiros M., Cambau E. (2020). Antimicrobial Susceptibility Testing
of Mycobacterium Tuberculosis Complex Isolates – the EUCAST
Broth Microdilution Reference Method for MIC Determination. Clin. Microbiol. Infect..

[21] EUCAST eucast: AST of yeasts; https://www.eucast.org/astoffungi/methodsinantifungalsusceptibilitytesting/susceptibility_testing_of_yeasts. (accessed 2025–06–26).

[22] EUCAST eucast: AST of moulds; https://www.eucast.org/astoffungi/methodsinantifungalsusceptibilitytesting/ast_of_moulds. (accessed 2025–06–26).

[23] Jawad B., Poudel L., Podgornik R., Steinmetz N. F., Ching W.-Y. (2019). Molecular Mechanism and Binding Free Energy of Doxorubicin
Intercalation in DNA. Phys. Chem. Chem. Phys..

[24] Pushkaran A. C., Arabi A. A. (2026). Ensemble
Molecular Dynamics for Predicting Binding
Energies of DNA Intercalators. RSC Adv..

[25] Subirana J.
A., Soler-López M. (2003). Cations as
Hydrogen Bond Donors: A View of Electrostatic
Interactions in DNA. Annu. Rev. Biophys. Biomol.
Struct..

[26] Sivakova S., Rowan S. J. (2005). Nucleobases as Supramolecular
Motifs. Chem. Soc. Rev..

[27] Martin M. T., Bessas N. C., Llontop C. A. P., Sgro G. G., Da Silva R. S. (2026). Dual HSA
and DNA Affinity of a Free-Base Porphyrin Nitro-Ruthenium­(II) Complex:
Spectroscopic Evaluation and Photocleavage Studies. J. Inorg. Biochem..

[28] Ye C.-Q., Zhou L.-W., Fan C.-B., Dai G.-L., Wang X.-M., Tao X.-T., Tang P.-Y., Su W.-M. (2019). Aggregation-Induced
Ultraviolet Emission Enhancement and the Electroluminescence Based
on New Phenanthrene Derivatives. ChemistrySelect.

[29] Żurek-Biesiada D., Waligórski P., Dobrucki J. W. (2014). UV-Induced Spectral Shift and Protonation
of DNA Fluorescent Dye Hoechst 33258. J. Fluoresc..

[30] Wang Y., Schellenberg H., Walhorn V., Toensing K., Anselmetti D. (2017). Binding Mechanism
of Fluorescent Dyes to DNA Characterized by Magnetic Tweezers. Mater. Today: Proc..

[31] Margetić D., Jadrijević-Mladar P., Brozovic A., Tumir L.-M. (2025). Guanidino-Aryl
Derivatives: Binding to DNA, RNA and G-Quadruplex Structure and Antimetabolic
Activity. Molecules.

[32] Gan F., Yang P., Liang J., Shen C., Crassous J., Qiu H. (2023). DNA-Induced Circularly
Polarized Luminescence of Helicene Racemates. Chirality.

[33] Sprecher C. A., Baase W. A., Johnson W. C. (1979). Conformation and Circular Dichroism
of DNA. Biopolymers.

[34] Honzawa S., Okubo H., Anzai S., Yamaguchi M., Tsumoto K., Kumagai I. (2002). Chiral Recognition
in the Binding
of Helicenediamine to Double Strand DNA: Interactions between Low
Molecular Weight Helical Compounds and a Helical Polymer. Bioorg. Med. Chem..

[35] Podsiedlik M., Markowicz-Piasecka M., Sikora J. (2020). Erythrocytes as Model Cells for Biocompatibility
Assessment, Cytotoxicity Screening of Xenobiotics and Drug Delivery. Chem.-Biol. Interact..

[36] Farag M. R., Alagawany M. (2018). Erythrocytes as a Biological Model for Screening of
Xenobiotics Toxicity. Chem.-Biol. Interact..

