# Supplementary material for: Design, Synthesis, and Evaluation of a Novel Phenanthrene Derivative as a Potential DNA Intercalator
Source: ACS Chem Biol. 2026 May 16;21(6):1434–42. doi: 10.1021/acschembio.6c00169 (PMC13288466; doi:10.1021/acschembio.6c00169)
Supplement: Supplementary file 1 [file cb6c00169_si_001.pdf]

Supplementary Materials: Design, Synthesis, and Evaluation of a Novel Phenanthrene-Derivative as a Potential DNA Intercalator

Ghada Bouz[1,2†*], Giulia Quaglia[3†], Loredana Latterini[3], Pavel Barta[4], Ondřej Janďourek[4], Klára Konečná[4], Jan Ősterreicher[4], Lieve Naesens[5], Leentje Persoons[5], Jan Storch[1] and Illia Panov[1*]

[1]Research Group of Advanced Materials and Organic Synthesis, Institute of Chemical Process Fundamentals of the Czech Academy of Sciences, Rozvojova 1/135, 165 00 Prague 6, Czech Republic; storchj@icpf.cas.cz (J.S.)
[2]Faculty of Pharmacy, University Business Academy, Heroja Pinkija 4, 21101 Novi Sad, Serbia
[3]Nano4Light Lab, Department of Chemistry, Biology and Biotechnology, University of Perugia, Via Elce di Sotto 8, 06123, Perugia, Italy ; giulia.quaglia@unipg.it (G.Q.); loredana.latterini@unipg.it (L.L.)
[4]Faculty of Pharmacy in Hradec Králové, Charles University, Hradec Králové, Czech Republic; bartp7aa@faf.cuni.cz (P.B.); jando6aa@faf.cuni.cz (O.J.); konecna@faf.cuni.cz (K.K.); sterreij@faf.cuni.cz (J.O.)
[5]KU Leuven, Department of Microbiology, Immunology and Transplantation, Rega Institute, Leuven, Belgium; lieve.naesens@kuleuven.be (L.N.); leentje.persoons@kuleuven.be (L.P.)

[†]These authors contributed equally

[*]Correspondence: ghada.bouz@ffns.ac.rs; panov@icpf.cas.cz

Photophysical Characterization

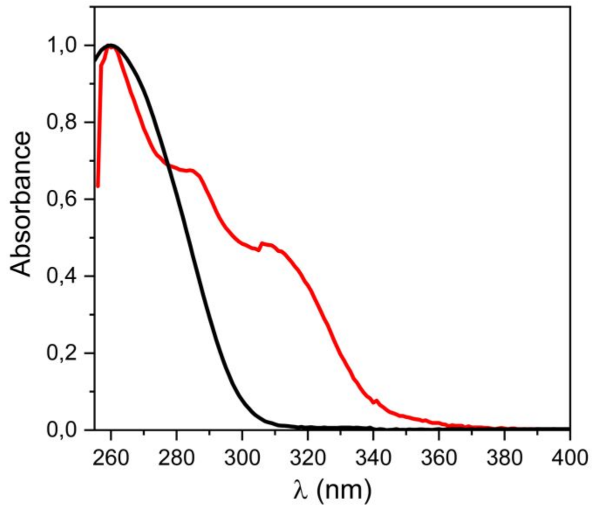

Figure S1: *Absorption spectra of PHE-CYT-3,6-TFA in DMSO 5μM (red line) and DNA 8μM aqueous solution (black line).*

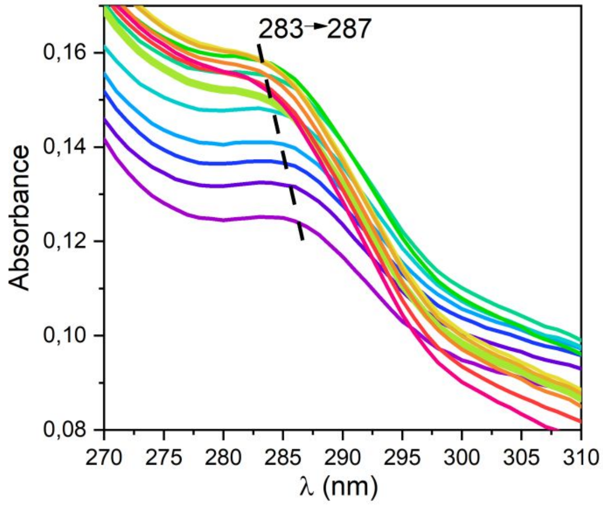

Figure S2: *Absorption spectra of PHE-CYT-3,6-TFA in DMSO measured with different concentrations of DNA aqueous solution (range: 0.019–3.211 µg/mL).*

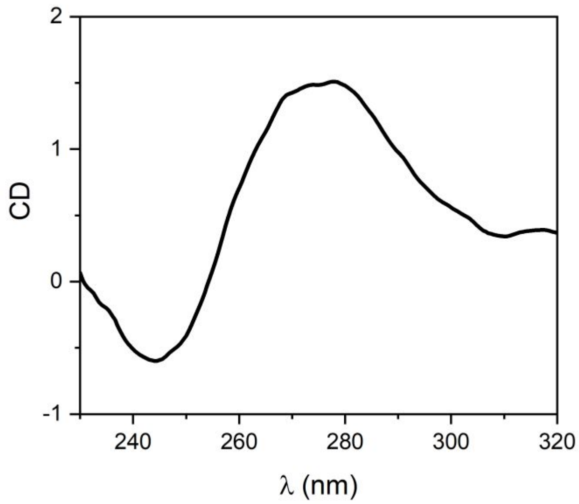

Figure S3: *Circular dichroism spectrum of DNA water solution.*

In vitro cytotoxicity against A498, HK-2, PC-3, SK-OV-3, and U-87 MG cell lines

The used cell cultures (except HepG2 cell line) are commercially available from American Type Culture Collection (ATCC, Manassas, VA, USA). Cell lines were tested on mycoplasma contamination upon their delivery. The human hepatocellular liver carcinoma cell line HepG2 (ECACC, Salisbury, UK) was cultured in Minimum Essentials Eagle Medium (denoted MEM) supplemented with 10% fetal bovine serum (PAA Laboratories, Pasching, Austria), 1% L-glutamine solution and non-essential amino acid solution. Human epithelial kidney carcinoma cells A-498 (ATCC HBT-44) were cultured in Dulbecco's Modified Eagles Medium Low Glucose (denoted DMEM Low Glucose) supplemented with fetal bovine serum (10%),

L-glutamine (1%), and penicillin/streptomycin (1%). Human proximal tubule kidney cells HK-2 (ATCC CRL-2190) and human glioblastoma cells U-87 MG (ATCC HTB-14) were cultured in Dulbecco's Modified Eagles Medium High Glucose (denoted DMEM High Glucose) supplemented with fetal bovine serum (10%), Non-Essential Amino Acids (1%), and penicillin/streptomycin (1%). Human prostate adenocarcinoma cells PC-3 (ATCC CRL-1435) were cultured in Kaighn's Modification of Ham's F-12 Medium (denoted F-12K Medium; Gibco, ThermoFisher Scientific) supplemented with fetal bovine serum (10%) and penicillin/streptomycin (1%). Human ovary adenocarcinoma cells SK-OV-3 (ATCC HTB-77) were cultured in McCoy's 5A Medium (Gibco, ThermoFisher Scientific) supplemented with fetal bovine serum (10%) and penicillin/streptomycin (1%). Used media and supplements were purchased from Merck (USA) unless otherwise stated. All cell lines were cultured at 37 °C, 5% CO_2 in a humidified atmosphere and grown to confluence.

On the experimental day, cells were seeded into a 96-well plate in the quantity 1.0×10^4 cells/100 µL of cell media per well. After 24h incubation at 37 °C, cells were treated with the tested compounds in the final volume of 100 µL containing 1% DMSO (*v*/*v*). Cells were incubated with the tested compounds at the following concentrations 1, 5, 10, 25, 50, 100, 250, 500, and 1000 µM for 24 h. Controls were treated with cell culture medium only (100% viability) and with 1% DMSO (*v*/*v*, the control of no cytotoxic effect of 1% DMSO) dissolved in appropriate cell culture medium at 37 °C for 24 h. The absolute mortality of cells was also analyzed with 10% DMSO (*v/v*) dissolved in appropriate cell culture medium at 37 °C for 24 h. Controls and compound concentrations were made in triplicates. When the incubation was over, the reagent from the kit CellTiter 96 AQueous One Solution Cell Proliferation Assay (CellTiter 96; PROMEGA, Fitchburg, USA) was added. After 2h incubation at 37 °C, absorbance in each sample well was recorded at 490 nm (TECAN, Infinita M200, Austria).

The 50% inhibitory concentrations (IC_{50}) values were determined from the dose-response curves in GraphPad Prism 10 (GraphPad Software, Boston, MA USA). Viability (%) was plotted as a function of concentration (log values), fitted to a sigmoidal curve, in which the half maximal inhibitory concentration value was determined representing the concentration of a compound required for 50% inhibition.

In vitro antibacterial activity screening

The microdilution broth method was performed according to EUCAST (The European Committee on Antimicrobial Susceptibility Testing) instructions [1] with slight modifications. Eight testing bacterial strains (four G+ and four G-) were purchased from the Czech Collection of Microorganisms (CCM, Brno, Czech Republic) or from the German Collection of Microorganisms and Cell Cultures (DSM, Braunschweig, Germany): *Staphylococcus aureus* subsp. *aureus* CCM 4223 (ATCC 29213), methicillin-resistant *Staphylococcus aureus* subsp. *aureus* (MRSA) CCM 4750 (ATCC 43300), *Staphylococcus epidermidis* CCM 4418 (ATCC 12228), *Enterococcus faecalis* CCM 4224 (ATCC 29212), *Escherichia coli* CCM 3954 (ATCC 25922), *Klebsiella pneumoniae* CCM 4415 (ATCC 10031), *Acinetobacter baumannii* DSM 30007 (ATCC 19606), *Pseudomonas aeruginosa* CCM 3955 (ATCC 27853). The cultivation was done in Cation-adjusted Mueller-Hinton broth (CAMHB, M-H 2 Broth, Merck, Darmstadt, Germany) at 35 ± 2 °C. Tested compounds were dissolved in DMSO (Merck) to produce stock solutions. The final concentration of DMSO in the cultivation medium did not exceed 1% (v/v) of the total solution composition and did not affect the growth of bacteria. Positive growth controls consisted of test microbe solely, negative growth controls consisted of cultivation medium. Antibacterial activity was expressed as minimum inhibitory concentration (MIC, in µM) after 24 and 48 h of static incubation in the dark and humidified atmosphere, at 35 ± 2 °C. Visual

inspection was used for MIC endpoint evaluation. The internal quality standards of gentamicin and ciprofloxacin (both from Merck) were involved in assay.

In vitro antimycobacterial activity screening

The antimycobacterial assay was performed with rapidly growing *Mycolicibacterium smegmatis* DSM 43465 (ATCC 607), *Mycolicibacterium aurum* DSM 43999 (ATCC 23366), and non-tuberculous mycobacteria, namely *Mycobacterium avium* DSM 44156 (ATCC 25291), *Mycobacterium kansasii* DSM 44162 (ATCC 12478) from German Collection of Microorganisms and Cell Cultures (Braunschweig, Germany), and with an avirulent strain of *Mycobacterium tuberculosis* H37Ra ITM-M006710 (ATCC 9431) from Belgian Co-ordinated Collections of Micro-organisms (Antwerp, Belgium). The technique used for activity determination was the microdilution broth panel method using 96-well microtitration plates. The liquid culture medium used in all assays was Middlebrook 7H9 broth (Merck, Darmstadt, Germany) enriched with 0.4% glycerol (Merck, Darmstadt, Germany) and 10% Middlebrook OADC growth supplement (Himedia, Mumbai, India). The mycobacterial strains were cultured on supplemented Middlebrook 7H9 agar. The final density of starting inoculum was adjusted to 1.0 according to the McFarland scale and diluted in the ratio of either 1:20 (for rapidly growing mycobacteria) or 1:10 (for slow growing mycobacteria) with broth.

The tested compounds were dissolved in DMSO (Merck, Darmstadt, Germany), then Middlebrook broth was added to obtain a concentration of 2000 µg/mL. The standards used for internal quality control were isoniazid (INH), rifampicin (RIF), and ciprofloxacin (CIP) (Merck, Darmstadt, Germany). Final concentrations were reached by binary dilution and addition of mycobacterial suspension and were set as 500, 250, 125, 62.5, 31.25, 15.625, 7.81, and 3.91 µg/mL. Isoniazid was diluted in the range 500–3.91 µg/mL for screening against rapidly growing mycobacteria, 2000–15.625 µg/mL for *M. avium*, 50–0.39 µg/mL for *M. kansasii,* and 1-0.0078 µg/mL for *M. tuberculosis*. Rifampicin final concentrations ranged from 50 to 0.39 µg/mL for rapidly growing mycobacteria and *M. avium*, and from 0.1 to 0.00078 µg/mL for *M. tuberculosis* and *M. kansasii*. Ciprofloxacin was used for screening antimycobacterial activity with final concentrations of 1, 0.5, 0.25, 0.125, 0.0625, 0.0313, 0.0156, 0.0078 µg/mL. The final concentration of DMSO did not exceed 2.5% (v/v) and did not affect the growth of all strains. Positive (broth, DMSO, bacteria) and negative (broth, DMSO) growth controls were included. Plates containing slow-growing mycobacteria were sealed with polyester adhesive film and all plates were incubated in the dark at 37°C without agitation. The addition of a 0.01% solution of resazurin sodium salt followed after 48 hours of incubation for *M. smegmatis*, after 72 hours for *M. aurum,* after 96 hours for *M. avium* and *M. kansasii*, and after 120 hours for *M. tuberculosis*. Microtitration panels were then incubated for a further 2.5 hours to determine the activity against *M. smegmatis*, 4 hours for *M. aurum,* 6 hours for *M. avium* and *M. kansasii*, and 18 hours for *M. tuberculosis*. The antimycobacterial activity was expressed as minimal inhibition concentration (MIC) and the value was read on the basis of stain colour change (blue colour – active compound; pink colour – inactive compound). All experiments were conducted in duplicates.

In vitro antifungal activity screening

Antifungal activity evaluation was performed using a microdilution broth method according to EUCAST instructions [2,3] with slight modifications. Eight fungal strains (four yeasts and four molds) were used for antifungal activity screening, namely: *Candida albicans* CCM 8320 (ATCC 24433), *Candida krusei* CCM 8271 (ATCC 6258), *Candida parapsilosis* CCM 8260 (ATCC 22019), *Candida tropicalis* CCM 8264 (ATCC 750), *Aspergillus fumigatus* ATCC 204305, *Aspergillus flavus* CCM 8363, *Lichtheimia corymbifera* CCM 8077, and

Trichophyton interdigitale CCM 8377 (ATCC 9533). Tested strains were purchased from the Czech Collection of Microorganisms (CCM, Brno, Czech Republic) or from the American Type Collection Cultures (ATCC, Manassas, VA, USA).

Tested compounds were dissolved in DMSO and diluted in a two-fold manner with RPMI 1640 medium, containing L-glutamine and 2% glucose, buffered to pH 7.0 with MOPS (3-morpholinopropane-1-sulfonic acid). The final concentration of DMSO in the testing medium did not exceed 1% (v/v) of the total solution composition. Static incubation was performed in the dark and in a humid atmosphere, at 35 ± 2 °C, for 24 and 48 h (72 and 120 h for *Trichophyton interdigitale*, respectively). Positive growth controls consisted of test microbe solely, while negative growth controls consisted of cultivation medium and DMSO. Visual inspection and metabolic activity indicator, Alamar Blue (ThermoFisher Scientific, USA), were used for MIC endpoint evaluation. The internal quality standards, amphotericin B (Merck) and voriconazole (Toronto Research Chemicals, CA) were involved in assays (for IC_{50}, IC_{90}, MIC of standards, see below).

Employment of *ex vivo* human red blood cell hemolysis assay

Blood samples acquired from human volunteers were centrifuged (1000×g, 10 min), supernatants were discarded, and the pellets were washed three times with Hartmann's solution. The final pellet was diluted 1:7 (v/v) with Hartmann's solution. Then, 0.5 mL of cell suspensions were incubated with PHE-CYT-3,6-TFA, at the final concentrations corresponding to the range from 3,906 to 1000 μl. The mixture was kept at 37°C for 1h. After incubation, the cell suspension was centrifuged, and the supernatant was carefully collected. The amount of hemoglobin, released from red blood cells (RBC) into the supernatant, was monitored by measuring the absorbance at 405 nm [4,5] with a spectrophotometer (Synergy HTX Multi-mode reader, BioTek, USA). The negative control (RBC in Hartmann's solution only) and positive controls (RBC in Hartmann's solution, sonicated by ultrasonic needle (Ultrasonic Processor UP100H, Hielscher Ultrasonic, Germany) for 1 min), were included as well. The experiment was designed according to the procedures reported in 22,23. Statistical analysis was performed using Graph-Prism software version 10.0.0 (GraphPad Software, Inc., USA). Data from *ex vivo* human red blood cell hemolysis assay were subjected to one-way ANOVA test (Friedman test), and *p*-value < 0.05 was accepted as statistically significant. Data are present as the mean ± standard error of the mean (SEM).

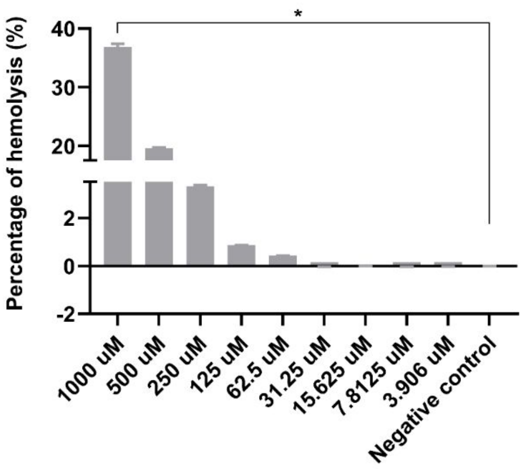

Figure S4*: Hemolysis assay. The impact of different concentrations of PHE-CYT-3,6-TFA on human red blood cells (RBC) is presented as the percentage of hemolysis. Negative controls (RBC in Hartmann's solution only) and positive controls (RBC in Hartmann's solution, sonicated by ultrasonic needle) were employed for the calculation of the percentage of hemolysis. Statistical significance was tested with one-way ANOVA, and p-value < 0.05 was accepted as statistically significant. The values shown are means of hexaplicates ± SEM.*

References

1. Hasselmann, C.; European Soc Clinical, M. Determination of minimum inhibitory concentrations (MICs) of antibacterial agents by broth dilution. *Clinical Microbiology and Infection* **2003**, *9*.
2. EUCAST DEFINITIVE DOCUMENT E.DEF 7.3.2. Available online: https://www.eucast.org/astoffungi/methodsinantifungalsusceptibilitytesting/susceptibility_testing_of_yeasts/ (accessed on 20.12.2022).
3. EUCAST DEFINITIVE DOCUMENT E.DEF 9.4. Available online: https://www.eucast.org/astoffungi/methodsinantifungalsusceptibilitytesting/ast_of_moulds (accessed on 20.12.2022).
4. Saebo IP, Bjoras M, Franzyk H, Helgesen E, Booth JA. Optimization of the Hemolysis Assay for the Assessment of Cytotoxicity. Int J Mol Sci. 2023;24(3).
5. Boehm D, Bell A. Simply red: A novel spectrophotometric erythroid proliferation assay as a tool for erythropoiesis and erythrotoxicity studies. Biotechnol Rep (Amst). 2014;4:34-41.
